# Auxotrophic *Actinobacillus pleurpneumoniae* grows in multispecies biofilms without the need for nicotinamide-adenine dinucleotide (NAD) supplementation

**DOI:** 10.1186/s12866-016-0742-3

**Published:** 2016-06-27

**Authors:** Abraham Loera-Muro, Mario Jacques, Francisco J. Avelar-González, Josée Labrie, Yannick D. N. Tremblay, Ricardo Oropeza-Navarro, Alma L. Guerrero-Barrera

**Affiliations:** Centro de Ciencias Básicas, Universidad Autónoma de Aguascalientes, Aguascalientes, Ags. Mexico 20131; Groupe de recherche sur la maladies infectieuses du porc, Faculté de médecine vétérinaire, Université de Montréal, St-Hyacinthe, Québec J2S 7C6 Canada; Departamento de Microbiología Molecular, Instituto de Biotecnología, Universidad Nacional Autónoma de México, Cuernavaca, Morelos Mexico 62260; Laboratorio de Biología Celular y Tisular, Departamento de Morfología, Centro de Ciencias Básicas, Universidad Autónoma de Aguascalientes, Aguascalientes, Ags. Mexico 20131

**Keywords:** Biofilms, *Actinobacillus pleuropneumoniae*, *Streptococcus suis*, *Bordetella bronchiseptica*, *Pasteurella multocida*, *Staphylococcus aureus*, *Escherichia coli*, Pyridine compounds

## Abstract

**Background:**

*Actinobacillus pleuropneumoniae* is the etiologic agent of porcine contagious pleuropneumonia, which causes important worldwide economic losses in the swine industry. Several respiratory tract infections are associated with biofilm formation, and *A. pleuropneumoniae* has the ability to form biofilms in vitro. Biofilms are structured communities of bacterial cells enclosed in a self-produced polymer matrix that are attached to an abiotic or biotic surface. Virtually all bacteria can grow as a biofilm, and multi-species biofilms are the most common form of microbial growth in nature. The goal of this study was to determine the ability of *A. pleuropneumoniae* to form multi-species biofilms with other bacteria frequently founded in pig farms, in the absence of pyridine compounds (nicotinamide mononucleotide [NMN], nicotinamide riboside [NR] or nicotinamide adenine dinucleotide [NAD]) that are essential for the growth of *A. pleuropneumoniae*.

**Results:**

For the biofilm assay, strain 719, a field isolate of *A. pleuropneumoniae* serovar 1, was mixed with swine isolates of *Streptococcus suis*, *Bordetella bronchiseptica*, *Pasteurella multocida*, *Staphylococcus aureus* or *Escherichia coli*, and deposited in 96-well microtiter plates. Based on the CFU results, *A. pleuropneumoniae* was able to grow with every species tested in the absence of pyridine compounds in the culture media. Interestingly, *A. pleuropneumoniae* was also able to form strong biofilms when mixed with *S. suis*, *B. bronchiseptica* or *S. aureus.* In the presence of *E. coli*, *A. pleuropneumoniae* only formed a weak biofilm. The live and dead populations, and the matrix composition of multi-species biofilms were also characterized using fluorescent markers and enzyme treatments. The results indicated that poly-*N*-acetyl-glucosamine remains the primary component responsible for the biofilm structure.

**Conclusions:**

In conclusion, *A. pleuropneumoniae* apparently is able to satisfy the requirement of pyridine compounds through of other swine pathogens by cross-feeding, which enables *A. pleuropneumoniae* to grow and form multi-species biofilms.

**Electronic supplementary material:**

The online version of this article (doi:10.1186/s12866-016-0742-3) contains supplementary material, which is available to authorized users.

## Background

*Actinobacillus pleuropneumoniae* is the etiologic agent of porcine contagious pleuropneumonia, an infectious disease of swine, which causes important economic losses in the pig industry worldwide [1–7]. *A. pleuropneumoniae* is a Gram-negative bacteria belonging to the *Pasteurellaceae* family [8–11]. Two biotypes have been described based on their dependence of nicotinamide adenine dinucleotide (NAD) and fifteen serovars are recognized [8, 12].

Many virulence factors have been reported in *A. pleuropneumoniae* [5, 6, 8, 13, 14], which include adhesion structures, such as type IV pilus [5, 15, 16], Flp pilus [3], and autotransporter adhesins [5], and biofilms formation [3, 5, 17]. Other swine pathogens, including *Bordetella bronchiseptica*, *Escherichia coli, Haemophilus parasuis*, *Salmonella typhymurium*, *Staphylococcus aureus*, and *Streptococcus suis*, can also form biofilms [9, 18].

Biofilms are structured communities of microorganisms, enclosed in a self-produced extracellular polymer matrix, attached to biological or non-biological surfaces [19–21]. Biofilms can be found in every ecosystem including natural, engineered and pathogenic settings. Growth as a biofilm is considered to be a protective mode that allows for survival in hostile environments [9]. Multi-species biofilms is likely the most prominent lifestyle of microorganisms outside the laboratory and the species composition of these biofilms will vary and be dependent on the environment [20, 22–25]. In multi-species biofilms, microorganisms will communicate, compete or cooperate to improve and ensure their survival and propagation [22, 24].

The goal of this study was to determine the ability of the swine respiratory pathogen *A. pleuropneumoniae* biotype 1, serovar 1 to form multi-species biofilms with other swine bacterial pathogens (*S. suis*, *B. bronchiseptica*, *P. multocida*, *S. aureus* or *E. coli*) that frequently are present in swine farms, in the absence of pyridine compounds (nicotinamide mononucleotide [NMN], nicotinamide riboside [NR] or nicotinamide adenine dinucleotide [NAD]). Pyridine compounds are essential for the growth of *A. pleuropneumoniae* biotype 1. The live and dead populations, and the matrix composition of multi-species biofilms were also characterized using fluorescent markers and enzyme treatments.

## Methods

### Bacterial strains

Bacterial strains selected for this study were as follows: *A. pleuropneumoniae* biotype 1/ serovar 1 strain 719, three bacterial species belonging to the porcine respiratory disease complex (PRDC; *S. suis* serovar 2 strain 735, *B. bronchiseptica* strain 276, and *Pasteurella multocida* D strain 1703), a *S. aureus* isolated previously from a healthy pig (strain 154 N) and an enterotoxigenic *E. coli* (ETEC) isolated previously from pig (strain ECL17608). All bacteria were grown on brain heart infusion agar plates (BHI; Oxoid Ltd, Basingstoke, Hampshire, UK) with supplementation of 15 μg/mL NAD for *A. pleuropneumoniae* and only BHI for all the other bacteria. Plates were incubated overnight at 37 ºC with 5 % CO_2_. A colony was transferred into 5 mL BHI (Oxoid) with 15 μg/mL NAD or without this supplementation, and incubated at 37 °C overnight with agitation. This overnight culture was used for the biofilm assays. In this study no animal was utilized, because all experiments were done in vitro.

### Multi-species biofilm assay

Multi-species biofilm assays were performed as described previously Labrie et al. [17] for single-species with some modifications (Table 1). Briefly, overnight cultures of *A. pleuropneumoniae*, *S. suis, B. bronchiseptica, P. multocida*, *E. coli* or *S. aureus* were diluted 1/100 in BHI with and without supplementation of NAD. Volumes of the dilution were aliquoted by triplicate in wells of a sterile 96-well microtiter plate (Costar^®^ 3599, Corning, NY, USA) using the following template as an example: 100 μL *A. pleuropneumoniae* in BHI-NAD + 100 μL *S. suis* in BHI-NAD, or 100 μL *A. pleuropneumoniae* in BHI + 100 μL *S. suis* in BHI. The same template was used for the following combination: *A. pleuropneumoniae* - *B. bronchiseptica*, *A. pleuropneumoniae* - *P. multocida*, *A. pleuropneumoniae* - *E. coli*, and *A. pleuropneumoniae* - *S. aureus*. For the triple-species biofilms, 50 μL of each species were aliquoted in the wells. The triple-species combinations were as followed: *A. pleuropneumoniae* - *S. suis* - *B. bronchiseptica*, *A. pleuropneumoniae* - *S. suis* - *E. coli*; and *A. pleuropneumoniae* - *B. bronchiseptica* - *E. coli*. For single species controls, 75 μL of a species dilution in BHI-NAD was mixed with 75 μL of BHI-NAD, or 75 μL of a species dilution in BHI was mixed with 75 μL of BHI. Wells containing sterile broth were used as negative controls. Following an incubation of 24 h at 37 °C, the wells were washed by immersion in water and excess water was removed by inverting plates onto a paper towel. Biofilms were then stained with 0.1 % (w/v) crystal violet for 2 min, washed once with distilled water, dried at 37 °C for 30 min, and then 100 μL of ethanol (70 %) were added to the wells. Absorbance was measured at 590 nm using a spectrophotometer (Powerwave, BioTek Instruments, Winooski, VT, USA).

### Colony Forming Unit (CFU) counts

Colony forming units (CFU) of *A. pleuropneumoniae* and the other bacteria from biofilms were counted, using selective growth media and colony morphology. Briefly, multi-species biofilms were prepared as described above, the biofilms were washed once with sterile MilliQ water (200 μL) and the biofilms were detached using micropipette [26]. The samples were then serially diluted in NaCl (0.85 %). Dilutions were plated on the following media: BHI and BHI-NAD for *A. pleuropneumoniae* - *B. bronchiseptica* and *A. pleuropneumoniae* - *E. coli*, blood agar and blood agar-NAD (15 μg/mL) for *A. pleuropneumoniae* - *S. suis* and *A. pleuropneumoniae* - *P. multocida*, BHI-NAD-crystal violet (1 μg/mL) and mannitol salt agar for *A. pleuropneumoniae* - *S. aureus* (all media from Oxoid). Plates were incubated overnight at 37 ºC with 5 % CO_2_. The colonies grown on these selective media plates were then identified by colony morphology and counted, allowing for an estimation of the relative bacterial composition of multi-species biofilms.

### Confocal laser scanning microscopy (CLSM)

To determine the composition of the biofilm matrix, multi-species biofilms were prepared as described above and stained with FilmTracer FM 1-43 (Invitrogen, Eugene, OR), FilmTracer LIVE⁄DEAD Biofilm Viability Kit (Invitrogen), Wheat Germ Agglutinin (WGA-Oregon Green 488, Molecular Probes; which binds *N-*acetyl-D-glucosamine [PGA] and *N*-acetylneuraminic acid residues), FilmTracerTM SYPRO® Ruby biofilm matrix stain (Molecular Probes; binds proteins) or BOBO^TM^-3 iodide (Molecular Probes; label extracellular DNA or eDNA) as prescribed by the manufacturer. After 30 min incubation at room temperature, the fluorescent marker solution was removed, and the biofilms were washed with water. The wells were then filled with 100 μL of water or PBS for WGA-stained biofilms. The stained biofilms were visualized by CLSM (FV1000 IX81; Olympus, Markham, ON, Canada) and images were acquired using Fluoview software (Olympus).

### Enzymatic treatments of multi-species biofilms

Assays were performed in order to determine the stability of biofilms to enzymatic degradation. The enzymatic treatment assays were performed as described previously Tremblay et al. [27]. Biofilms were prepared as described above and 50 μL of dispersin B (100 μg/mL in PBS; Kane Biotech Inc., Winnipeg, MB, Canada), 50 μL of DNase I (500 μg/mL in 150 mM NaCl, 1 mM CaCl_2_), or 50 μL of proteinase K (500 μg/mL in 50 mM Tris-HCl pH 7.5, 1 mM CaCl_2_) were added directly to the biofilms. Samples with dispersin B were incubated for 5 min at 37 °C and samples with proteins K or DNase I were incubated for 1 h at 37 °C. Control wells were treated with 50 μL of the buffer without the enzyme. Biofilms were washed and then stained with crystal violet and the absorbance was measured at 590 nm.

### Fluorescent in situ hybridization (FISH)

In order to confirm the presence of *A. pleuropneumoniae* and determine its distribution and localization in the biofilms, FISH was performed as described Loera-Muro et al. [7] and Jensen et al. [28] with modifications. Single-species biofilms of *A. pleuropneumoniae* strain 719 was used as a positive control. Single-species biofilms of *S. suis* strain 735 was used as a probe-specificity control, and was grown as described by Wu et al. [18].

Biofilms were formed on glass slides with flexiPERM^®^ (eight wells; Sarstedt, Nümbrecht, Germany), by placing a glass slide in a Petri dish. A volume (300 μL) of dilution 1/100 of *A. pleuropneumoniae* or *S. suis* culture were added to the wells for single-species biofilms and 150 μL dilution 1/100 of *A. pleuropneumoniae* and 150 μL of *S. suis* or *B. bronchiseptica* culture were added to wells for dual-species biofilms. The biofilms were incubated for 24 h at 37 °C with 5 % CO_2_. The slides were then air-dried (1 h at 37 °C) and gently flamed. The biofilms were dehydrated in 100 % alcohol for 30 min before hybridization. The hybridization was carried out at 45 °C with 40 mL of hybridization buffer (100 mM Tris-HCl [pH 7.2], 0.9 M NaCl, 0.1 % sodium dodecyl sulfate) and 200 ng of each probe (APXIVAN-Forward [GGG GAC GTA ACT CGG TGA TT] and APXIVAN-Reverse [GCT CAC CAA CGT TTG CTC] labelled with 633 Alexa Fluor) for 16 h in a slide rack in the dark. The samples were then washed one time in prewarmed (45 °C) hybridization buffer for 15 min and subsequently one time in prewarmed (45 °C) washing solution (100 mM Tris-HCl [pH 7.2], 0.9 M NaCl). Samples were then washed with water for 5 min. To localize every bacterial cells, biofilms were also stained with FilmTracerTM FM® 1-43 fluorescent marker (Molecular Probes) according to manufacturer’s instructions after the hybridization step. To stain with FM 1-43, biofilms were incubated for 30 min at room temperature in the dark and then washed with water for 10 min. The samples were then covered with ProLong Gold antifade reagent (Invitrogen). The labeled bacteria were visualized using a CLSM (FV1000 IX81; Olympus) and images were acquired using Fluoview software (Olympus).

### Statistical analysis

All the statistical significance (*p* value < 0.05) analyses of differences in biofilm phenotypes (mean optical density values) were determined by a paired, one-tailed *t*-test using GraphPad Prism version 4.0 (GraphPad Software, San Diego, CA, USA).

## Results

### Multi-species biofilms formation with NAD supplementation

**Fig. 1:**
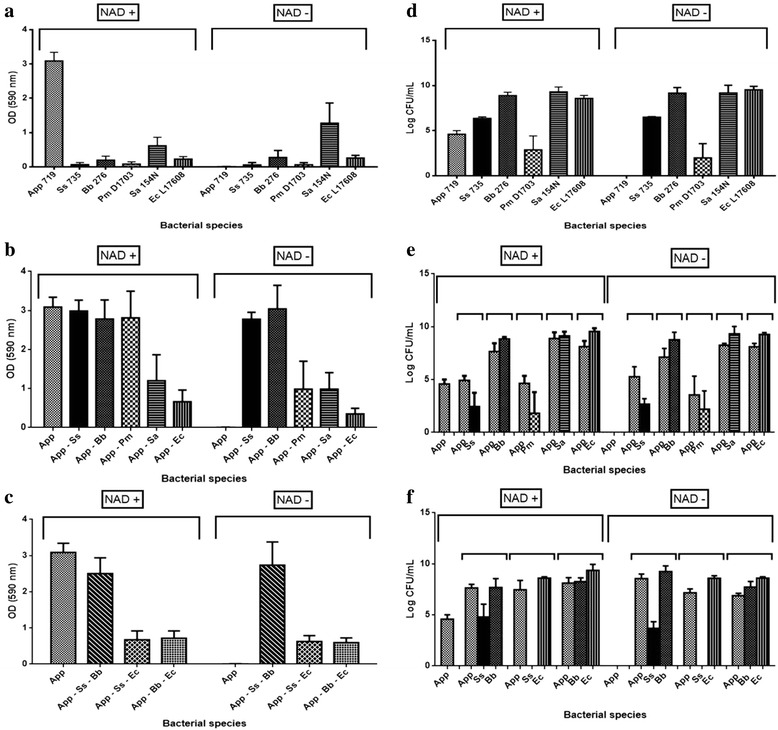
Multi-species biofilms formation by *A. pleuropneumoniae* with other swine pathogens. *A. pleuropneumoniae*, *S. suis*, *B. bronchiseptica*, *P. multocida*, *S. aureus* and *E. coli*. The biofilms grown as (**a**) single, (**b**) dual or (**c**) triple-species biofilms in BHI media with or without NAD, they were stained with crystal violet staining. The colony forming units (CFU) of *A. pleuropneumoniae* and the other bacteria were enumerated from multi-species biofilms grown in BHI media with or without NAD grown as (**d**) single, (**e**) two or (**f**) three-species biofilms. App: *A. pleuropneumoniae*; Ss: *S. suis*; Bb: *B. bronchiseptica*; Pm: *P. multocida*; Sa: *S. aureus*; Ec: *E. coli*

*A. pleuropneumoniae* serovar 1 strain 719, a field isolate, was used in a 96-well microtiter plate in multi-species biofilms assays. Under favorable growth conditions for *A. pleuropneumoniae* biofilm formation, bacteria belonging to PRCD (*S. suis*, *B. bronchiseptica* and *P. multocida*) did not reduce or inhibited biofilm formation by *A. pleuropneumoniae* (Fig. 1b)*.* By contrast, the presence of *E. coli* resulted in a decreased biofilm formation (Fig. 1b). In order to confirm that the other species were present in these biofilms, CFU of *A. pleuropneumoniae* and the other bacteria were determined (Fig. 1d – 1 f) and, in all cases, *A. pleuropneumoniae* and the other bacterial species were able to grow.

Because *E. coli* causes a decrease in the biofilm of *A. pleuropneumoniae*, a third species was added to the biofilm (*S. suis* or *B. bronchiseptica*) to determine if this negative effect presented by the *E. coli* dual-species biofilm was counteracted by the addition of a third bacteria species, forming a triple-species biofilm. This was done because in dual-species biofilm with *S. suis* and *B. bronchiseptica*, *A. pleuropneumoniae* was still able to form a strong biofilm (OD 2.505). However, *E. coli* still inhibited biofilm formation by *A. pleuropneumoniae* (OD 0.717, Fig. 1c) when a third species was added. Furthermore, *E. coli* prevented the growth of *S. suis,* as determined by CFU counts*.*

### Multi-species biofilms matrix composition with NAD supplementation

**Fig. 2:**
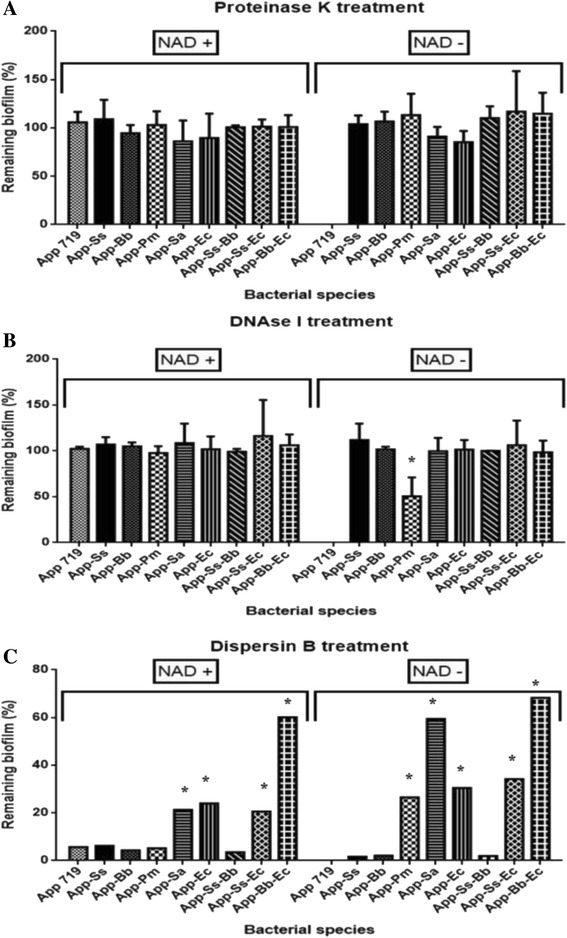
Effect of enzymatic treatment on multi-species biofilms. Dispersion by (**a**) proteinase K, (**b**) DNase I, and (**c**) dispersin B of multi-species biofilms formed by *A. pleuropneumoniae*, *S. suis*, *B. bronchiseptica*, *P. multocida*, *S. aureus* and *E. coli* grown in BHI media with or without NAD. App: *A. pleuropneumoniae*; Ss: *S. suis*; Bb: *B. bronchiseptica*; Pm: *P. multocida*; Sa: *S. aureus*; Ec: *E. coli*. * *p* < 0.05

To understand if the addition of a second species had impact on the composition and structure of the extracellular matrix of *A. pleuropneumoniae* biofilms, the biofilms were stained with fluorescent markers or treated with enzymes (Additional file 1:Figure S1). When PGA was labeling, some structural differences were observed. In most dual-species biofilms, PGA distribution appears to be in clusters or filament-like structures, whereas in *A. pleuropneumoniae* single-species biofilms, PGA distribution was homogeneous. Dispersin B, an enzyme that catalyzes the hydrolysis of linear polymers of N-acetyl-D-glucosamines, was able to disperse every multi-species biofilms (Fig. 2c). This suggests that PGA was the primary structural component seen in those multi-species biofilms. However, some significant increments in the resistance against the action of this enzyme in some multi-species biofilms were observed. These increments were observed in the two-species biofilms with *S. aureus* (21 %) and *E. coli* (24 %), and also in all the three-species biofilms with *E. coli* (increased between 20–68 %) (Fig. 2c).

When eDNA was labeled with BOBO^TM^-3 iodide, several differences were observed. With NAD supplementation, increases of eDNA were observed in the two-species biofilms formed with *B. bronchiseptica*, *P. multocida*, *S. aureus* and *E. coli*; and also in the three-species biofilms with *S. suis* and *B. bronchiseptica* (Additional file 1: Figure S1). Moreover, proteins not showed significant changes in most two-species biofilms, when they compared with the *A. pleuropneumoniae* mono-species biofilms. Only in the biofilms of *A. pleuropneumoniae - S. suis*, and in all biofilms with *E. coli* (two and three-species) the proteins composition were appreciated slightly lower. However, when they were treated with proteinase K and DNAse I, the biofilms remained attached to the surface indicating that proteins and eDNA do not participate in the integrity of the biofilms (Fig. 2a and b).

The labeling with FilmTracer FM 1-43, which inserts into the surface membrane on all bacteria, helped to evaluate the morphology of the biofilms. The morphology of two-species biofilms showed low changes, compared with the *A. pleuropneumoniae* mono-species biofilms. Moreover, in the three-species biofilms formed by *A. pleuropneumoniae, S. suis* and *B. bronchiseptica*, there was an increase in the occurrence of clusters, showed by fluorescence enhancement (Additional file 1: Figure S1).

When the composition of live/dead cells was evaluated in multispecies biofilms several changes were observed. Alive cells labeled with Syto9 were increased when biofilms of *A. pleuropneumoniae* were performed with *B. bronchiseptica*; with *S. aureus* or with *E. coli*, the same changes were observed also in three species biofilms. These results correlated also with CFU number detected in these multi-species biofilm when they were compared with the *A. pleuropneumoniae* mono-species biofilms.

Finally, dead cells were labeled with propidium iodide, and two scenarios were founded: the first was an increment of dead bacteria in biofilms of *A. pleuropneumoniae* - *B. bronchiseptica*, *A. pleuropneumoniae* - *P. multocida* and in the three-species *A. pleuropneumoniae* - *B. bronchiseptica* - *S. suis*. The second was the decreased amount of dead bacteria in multi-species biofilms of *A. pleuropneumoniae* with *S. aureus* and *E. coli*. In the first three cases, this increment can be related to the amount of eDNA observed, which also increases. In the last cases, also an increment is observed in the amount of eDNA. More studies about the specific compounds present in the extracellular matrix of these biofilms are necessary to understand which changes are produced for the presence the other species in biofilms with *A. pleuropneumoniae*.

### Multi-species biofilms formation without NAD supplementation

For growth in vitro, *A. pleuropneumoniae* biotype 1 requires the addition of a pyridine source, which includes NMN, NR or NAD, in the growth media. It is well established that *A. pleuropneumoniae* can growth without NAD supplementation in presence of *S. aureus* [29]. To test if *A. pleuropneumoniae* could grow and satisfy the requirements of pyridine compounds through of other swine pathogen species and also form biofilms, the multi-species biofilms assays were also performed in the absence of NAD. Based on OD and CFU results, *A. pleuropneumoniae* was able to grow, form multi-species biofilms and satisfy the requirements of pyridine compounds with every bacterial species tested in this study. *A. pleuropneumoniae* was able to form strong biofilms with the swine respiratory pathogens *S. suis* (OD 2.762) and *B. bronchiseptica* (OD 3.042). *A. pleuropneumoniae* formed a biofilm with the nasal isolate, *S. aureus* that was similar to the one with NAD supplement; however, this dual-species biofilm did not reach the same level as the *A. pleuropneumoniae* single-species biofilm. *A. pleuropneumoniae - P. multocida* dual-species biofilms were significant weaker (OD 0.987, *p* < 0.001) than with NAD supplementation (OD 2.811). As observed before, *A. pleuropneumoniae* formed a weak biofilm in the presence *E. coli* (OD 0.349).

Based on the CFU counts, the number of cells and species ratio did not change in the presence or absence of NAD (Fig. 1e). Thus, the absence of NAD did not have a significant impact species composition of the mix biofilms (Fig. 1e). This suggests that the absence of NAD was not a limiting factor for *A. pleuropneumoniae* growth in the presence of these bacteria species.

The proportions of live/dead bacteria in the multi-species biofilms were also observed with live/dead staining and CLSM. In the presence of *P. multocida, E. coli* or *S. aureus*, the dead bacteria population decreased when compared to the *A. pleuropneumoniae* control in BHI-NAD.

Regarding with the triple-species biofilms, again *A. pleuropneumoniae* was able to form strong biofilms with *S. suis* - *B. bronchiseptica* (OD 2.737) and weak biofilms when *E. coli* was present (OD 0.594, Fig. 1c). Furthermore, the species ratios were similar to the in the triple-species biofilms formed with NAD supplementation (Fig. 1f).

### Multi-species biofilms matrix composition without NAD supplementation

**Fig. 3:**
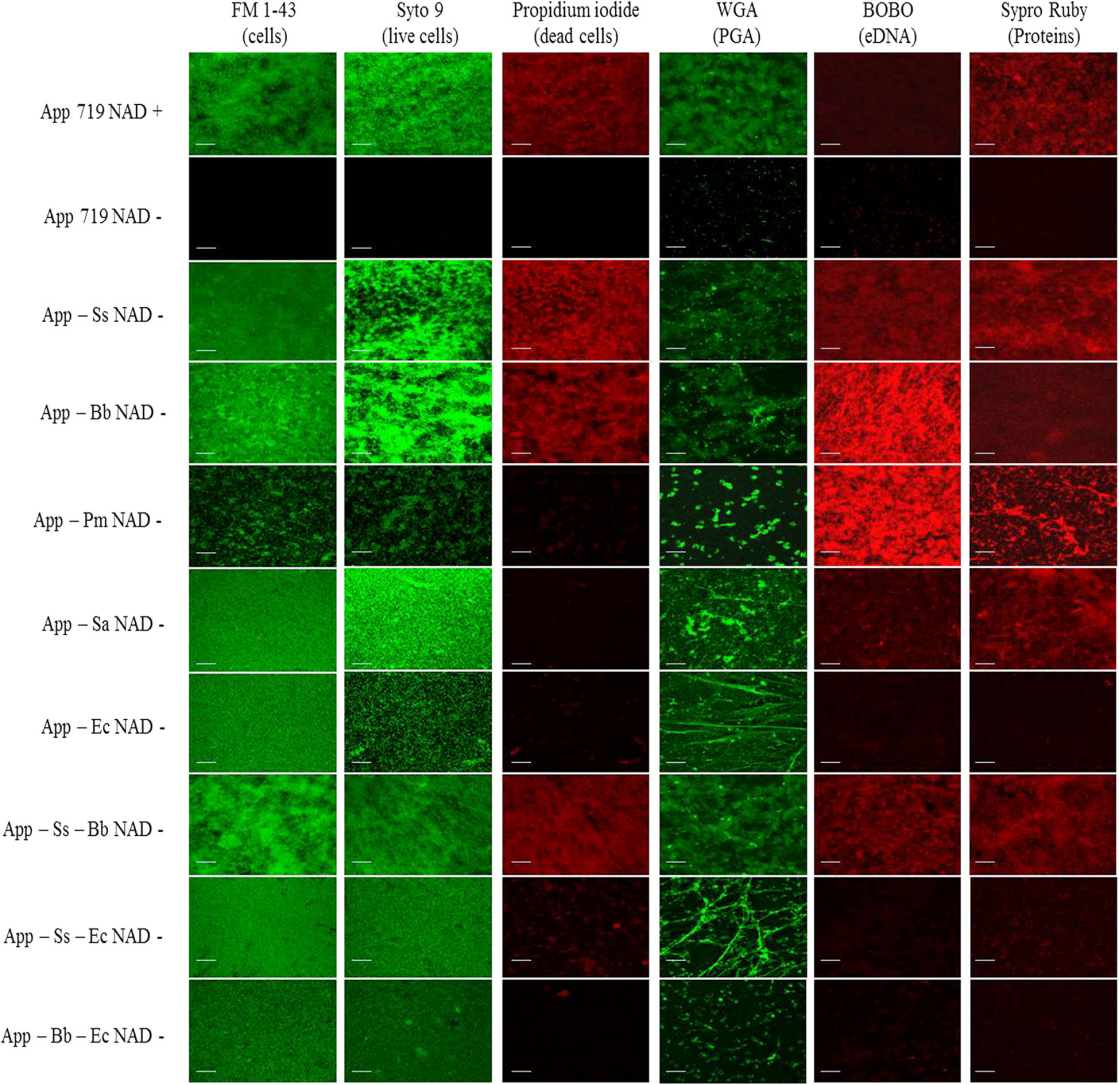
CLSM of multi-species biofilms of *A. pleuropneumoniae* with other swine pathogens without NAD supplementation. *A. pleuropneumoniae*, *S. suis*, *B. bronchiseptica*, *P. multocida*, *S. aureus* and *E. coli* grown as single, dual or triple-species biofilms in BHI media without NAD stained with FM 1-43, SYTO 9, propidium iodide, wheat-germ agglutinin (WGA)-Oregon green, BOBO-3, and SYPRO Ruby (all from Invitrogen, Eugene, OR). PGA: poly-*N*-acetylglucosamine; eDNA: extracellular DNA; App: *A. pleuropneumoniae*; Ss: *S. suis*; Bb: *B. bronchiseptica*; Pm: *P. multocida*; Sa: *S. aureus*; Ec: *E. coli*. Scale bar 30 μm

The composition of the extracellular matrix was characterized with fluorescent staining or enzymatic digestion (Fig. 2 and 3). Every multi-species biofilms were stained by WGA showing PGA in the biofilm. It was observed as clusters or with filament-like in some biofilms, PGA distribution was not homogenous in the multi-species biofilms. As was described before, all biofilms were sensitive to dispersin B treatment and this support that PGA is present in the biofilm; however, some biofilms were more resistant to the treatment. For example, 59 % and 75 % of the *A. pleuropneumoniae*-*S. aureus* biofilm and the *A. pleuropneumoniae* - *B. bronchiseptica* - *E. coli* biofilm remained after the treatment. This suggests that other components are important for the integrity of those biofilms.

For eDNA, an increase in the amount of stained eDNA was observed in the *A. pleuropneumoniae-B. bronchiseptica* and *A. pleuropneumoniae-P. multocida* biofilm (Fig. 3). Furthermore, the biofilm of *A. pleuropneumoniae-P. multocida* was sensitive to the treatment with DNase I and 50 % of the biofilm remained attached. Every other biofilms were resistant to DNase I treatment. This shows that eDNA is a structural component in the *A. pleuropneumoniae - P. multocida* biofilm but not for the other biofilms. With respect to proteins, an increase in staining was observed in the biofilms of *A. pleuropneumoniae* - *P. multocida.* However, the biofilm was resistant to proteinase K treatment as observed for the other multi-species biofilms.

Concluding, the analysis of the biofilm matrix indicates that PGA remains a major component responsible for the integrity of the biofilm. However, eDNA also play a role in the integrity of the *A. pleuropneumoniae* - *P. multocida* biofilm.

### Confirmation of the presence of *A. pleuropneumoniae* in multi-species biofilms by FISH

**Fig. 4:**
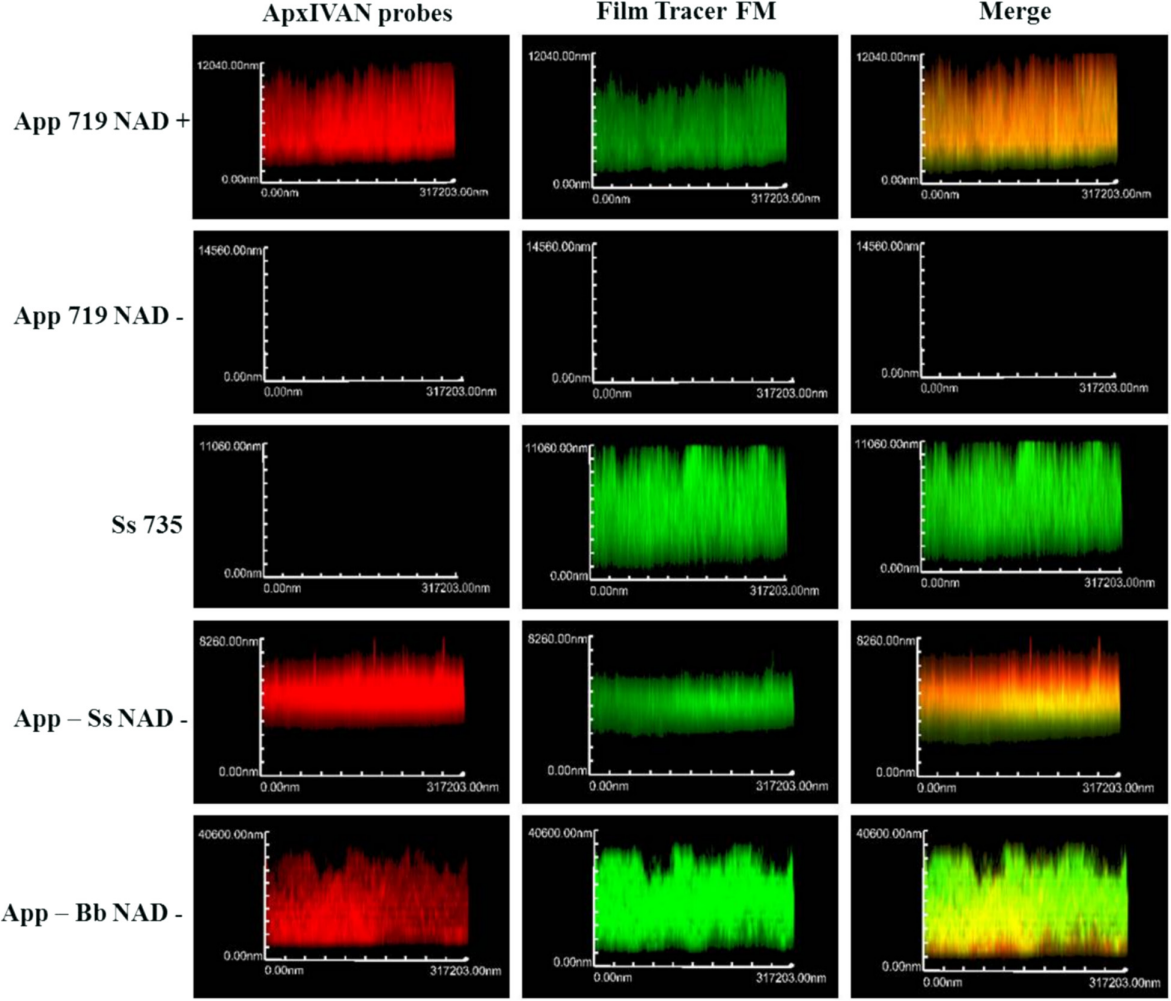
Confirmation of the presence of *A. pleuropneumoniae* in multi-species biofilms. Confirmation of *A. pleuropneumoniae* in the dual-species biofilm of *A. pleuropneumoniae* 719 and *S. suis* 735 or *B. bronchiseptica* 276 by FISH with an ApxIVAN-AlexaFluor 633 probe (red). Images of the X-Z plane of biofilm of single and dual-species biofilms grown in BHI with or without NAD. Bacterial cell in the biofilms were stained with FilmTracer ™ FM ® 1-43 (Molecular Probes) which are represented in green. Yellow represent the co-localization of both the ApxIVAN probe and the stain FM 1-43. App: *A. pleuropneumoniae*; Ss: *S. suis*; Bb: *B. bronchiseptica*

**Fig. 5:**
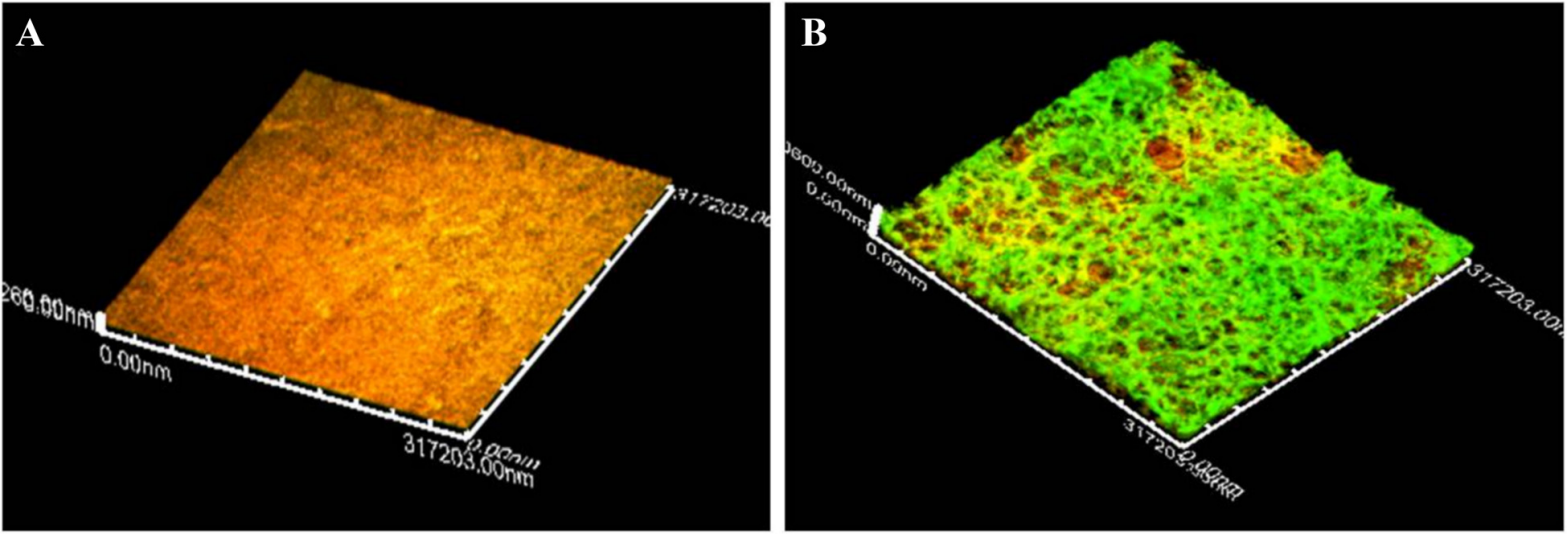
3D models of *A. pleuropneumoniae* in multi-species biofilms. Duals-species biofilms of (**a**) *A. pleuropneumoniae* 719 and *S. suis* 735 or (**b**) *A. pleuropneumoniae* 719 and *B. bronchiseptica* 276 detected with ApxIVAN-AlexaFluor 633 probe (red). Bacterial cell in the biofilms were stained with FilmTracer ™ FM ® 1-43 (Molecular Probes) which are represented in green. Yellow represent the co-localization of both the ApxIVAN probe and FM 1-43

To confirm the presence of *A. pleuropneumoniae* in these multi-species biofilms, FISH assays were performed for the *A. pleuropneumoniae*-*S suis* and *A. pleuropneumoniae* -*B. bronchiseptica* biofilms. In both cases, the presence of *A. pleuropneumoniae* was confirmed in the biofilms (Fig. 4). However, its distribution in the biofilms resulted different. For the *A. pleuropneumoniae*-*S. suis* biofilm, *A. pleuropneumoniae* appears in a layer on top of the biofilm and *S. suis* in the bottom of the same (Fig. 4 and 5a). For the *A. pleuropneumoniae*-*B. bronchiseptica* biofilm, *A. pleuropneumoniae* was mainly observed at the bottom of the biofilm whereas *B. bronchiseptica* was mainly on top of the biofilm (Fig. 4 and 5b).

## Discussion

This study demonstrates that *A. pleuropneumoniae* is able to form multi-species biofilms with other bacteria isolated from the swine respiratory tract. Importantly, *A. pleuropneumoniae* was able to form strong biofilms with other respiratory pathogen of swine belonging to PRDC (*S. suis*, *B. bronchiseptica* and *P. multocida*), and with a nasal isolate of *S. aureus* whereas *A. pleuropneumoniae* biofilm formation was decrease in the presence of the intestinal pathogen, *E. coli*. Furthermore, the key finding was that *A. pleuropneumoniae* could satisfy its requirements of pyridine compounds from other swine pathogens to grow and form biofilms. The fact that *A. pleuropneumoniae* was able to get pyridine compounds (probably NAD, NMN or NR [30, 31]) from other bacteria suggest that *A. pleuropneumoniae* could be using this strategy to form multi-species biofilms and persist in its host [8, 17] and/or to survive in the environment [7, 32].

Knowledge regarding the ability of swine respiratory pathogens, such as *A. pleuropneumoniae*, to form multi-species biofilms is limited and could provide important clues for processes during infection and persistence in the host and in the environment [7, 9]. Respiratory diseases in pigs have a polymicrobial nature [11, 33], but it is not yet established if porcine respiratory diseases could involve multi-species biofilms. However, it has been reported that several human and animal pathogens, such as *Pseudomonas aeruginosa*, *Stenotrophomonas maltophilia*, *Legionella pneumophila*, *Klebsiella pneumoniae, Fusobacterium nucleatum*, *Streptococcus mutans* and *E. coli*, can form multi-species biofilms, and these biofilms help them to increase their pathogenicity, resistance and their persistence in the environment [22, 24–26, 34–41]. The lower respiratory tract, has a limited essential nutrients supply for bacterial growth [8]. Although mammalian cells contain significant amounts of NAD(P)(H), the supply of pyridine nucleotides in extracellular fluids is probably quite low because several types of mammalian cells possess extrinsic NAD(P) + nucleosidases [30]. In response of this, *A. pleuropneumoniae* has developed a number of virulence mechanisms to overcome this lack, such as cell lysis that allows the release of nutrients into the surrounding environment [8]. Beside, multi-species biofilm formation by this bacterium with other porcine respiratory pathogens, such as *S. suis*, *B. bronchiseptica* and *P. multocida*; and other isolated bacteria from the porcine respiratory tract, among which *S. aureus* could be important either for chronic infections development or persistence, where *A. pleuropneumoniae* could be obtained these pyridine compounds from the other bacteria without causing further host damage; as described in several other diseases [42, 43]. Furthermore, multi-species biofilm formation could be allowing that *A. pleuropneumoniae* survive outside the host in the environment [7, 32]. More studies are needed to know the exact role of these multi-species communities and if it could play of role in the case of PRDC.

In *A. pleuropneumoniae*, biofilms formation over polystyrene microtiter plates depends on PGA production [9, 18, 27, 44] but protein and eDNA have also been reported [17]. PGA is the main component responsible for the multi-species biofilms integrity. However, some biofilms were less sensitive to dispersin B treatment suggesting that other matrix components are required for the multi-species biofilms integrity. Moreover, knowledge is limited regarding changes in the extracellular matrix composition when single-species biofilms become multi-species [45, 46]. Here, variations in the PGA distribution were observed in most multi-species biofilms with the presence of clusters or filament-like structures. Changes in the polysaccharide distribution could be related with the formation of small clusters by *A. pleuropneumoniae*, or by the production of additional polysaccharides by the secondary species, as *E. col*i*,* which produces cellulose to form biofilms [9]. Different polysaccharides production is supported by the fact that *E. coli* multi-species biofilms were resistant to the treatments with dispersin B, DNase I and proteinase K.

Interestingly, eDNA an important factor in multi-species biofilms [47, 48] was identified as a matrix component for the *A. pleuropneumoniae* - *P. multocida* dual-species when NAD was not supplemented. There was important increase in the eDNA staining, and the biofilm were sensitive to DNase I treatment. This was not observed for *A. pleuropneumoniae* single-species biofilms. Hathroubi et al., [49] recently report changes in the structure composition of the *A. pleuropneumoniae* extracellular matrix when exposed to sub-inhibitory concentrations (sub-MIC) of penicillin G, noting that eDNA play a structural role, they concluded that because sub-MIC of penicillin G induce cell death in a limited population, this allows the release of chromosomal DNA, which in turn could be used by the surviving bacteria to build the biofilm. Here, was observed a limited number of dead bacteria This change was only observed in the biofilm formed by *A. pleuropneumoniae* with *P. multocida* without NAD supplementation. More studies are necessaries to understand the importance of eDNA in multi-species biofilms.

Additionally, Disperin B was unable to completely segregate the multi-species biofilm formed by both bacteria. Indicating that *P. multocida* is actively participating in the structure of the extracellular matrix components. Unfortunately, little information exists about the ability of *P. multocida* to form biofilms and its extracellular matrix composition [50–52]. This highlights that fact multi-species biofilm composition depends directly on the species involved in the biofilm formation.

## Conclusion

In conclusion, our data show that *A. pleuropneumoniae* has the ability to form multi-species biofilms with respiratory porcine pathogens, *S. suis*, *B. bronchiseptica*, and *P. multocida*, and with other bacteria isolated from pigs. Furthermore, we report for the first time that *A. pleuropneumoniae* is able to satisfy the requirement of pyridine compounds through of other bacteria and this supported its growth and biofilm formation. Further research is needed to understand the pathway by which *A. pleuropneumoniae* can obtain its requirements of pyridine compounds, besides if these multi-species biofilm interactions are involved in the onset and/or development of swine respiratory diseases. Furthermore, the multi-species biofilm interactions could increase the resistance and/or persistence of *A. pleuropneumoniae* in its host, and in the environment.

## Abbrevations

NMN, nicotinamide mononucleotide; NR, nicotinamide riboside; NAD, nicotinamide adenine dinucleotide; BHI, brain heart infusion agar; CFU, colony forming units; PGA, *N-*acetyl-D-glucosamine; FISH, Fluorescent in situ hybridization

## Additional file

Additional file 1: Figure S1. CLSM of multi-species biofilms of *A. pleuropneumoniae* with the other swine pathogens with NAD supplementation. *A. pleuropneumoniae*, *S. suis*, *B. bronchiseptica*, *P. multocida*, *S. aureus* and *E. coli* grown in single, dual or triple-species biofilms in BHI media with NAD stained with FM 1-43, SYTO 9, propidium iodide, wheat-germ agglutinin (WGA)-Oregon green, BOBO-3, and SYPRO Ruby (all from Invitrogen, Eugene, OR). PGA: poly-*N*-acetylglucosamine; eDNA: extracellular DNA; App: *A. pleuropneumoniae*; Ss: *S. suis*; Bb: *B. bronchiseptica*; Pm: *P. multocida*; Sa: *S. aureus*; Ec: *E. coli*. Scale bar 30 μm (TIF 37984 kb)

## References

[1] Brockmeier S, Halbur P, Thacker, E. Porcine Respiratory Disease Complex. In: Brogden K, Guthmiller J, editors. Polymicrobial Diseases. ASM Press: American Society for Microbiology; 2002;231-58.

[2] Ramjeet M, Cox A, Hancock M, Mourez M, Labrie J (2008). Mutation in the LPS outer core biosynthesis gene, galU, affects LPS interaction with the RTX toxins ApxI and ApxII and cytolytic activity of *Actinobacillus pleuropneumoniae* serovar 1. Mol Microbiol.

[3] Auger E, Deslandes V, Ramjeet M, Contreras I, Nash J (2009). Host Pathogen Interactions of *Actinobacillus pleuropneumoniae* with Porcine Lung and Tracheal Epithelial Cells. Infect Immun.

[4] Buettner F, Konze S, Maas A, Gerlach G (2011). Proteomic and immunoproteomic characterization of a DIVA subunit vaccine against *Actinobacillus pleuropneumoniae*. Proteome Sci.

[5] Li L, Xu Z, Zhou Y, Li T, Sun L (2011). Analysis on *Actinobacillus pleuropneumoniae* LuxS regulated genes reveals pleiotropic roles of LuxS/AI-2 on biofilm formation, adhesion ability and iron metabolism. Microb Pathog.

[6] Li L, Xu Z, Zhou Y, Sun L, Liu Z (2012). Global Effects of Catecholamines on *Actinobacillus pleuropneumoniae* Gene Expression. PLoS ONE.

[7] Loera-Muro V, Jacques M, Tremblay Y, Avelar-González F, Loera-Muro A (2013). Detection of *Actinobacillus pleuropneumoniae* in drinking water from pig farms. Microbiology.

[8] Chiers K, De Waele T, Pasmans F, Ducatelle R, Haesebrouck F (2010). Virulence factors of *Actinobacillus pleuropneumoniae* involved in colonization, persistence and induction of lesions in its porcine host. Vet Res.

[9] Jacques M, Aragon V, Tremblay YD (2010). Biofilm formation in bacterial pathogens of veterinary importance. Anim Health Res Rev.

[10] Klitgaard K, Friis C, Jensen T, Angen Ø, Boye M (2012). Transcriptional Portrait of *Actinobacillus pleuropneumoniae* during Acute Disease – Potential Strategies for Survival and Persistence in the Host. PLoS ONE.

[11] Loera-Muro A, Avelar-González F, Loera-Muro V, Jacques M, Guerrero-Barrera A (2013). Presence of *Actinobacillus pleuropneumoniae, Streptococcus suis, Pasteurella multocida, Bordetella bronchiseptica, Haemophilus parasuis* and *Mycoplasma hyopneumoniae* in upper respiratory tract of swine in farms from Aguascalientes. Mexico OJAS.

[12] Perry MB, Angen O, Maclean LL, Lacouture S, Kokotovic B (2011). An atypical biotype I *Actinobacillus pleuropneumoniae* serovar 13 is present in North America. Vet Microbiol.

[13] Lone A, Deslandes V, Nash J, Jacques M, MacInnes J. *malT* knockout mutation invokes a stringent type gene-expression profile in *Actinobacillus pleuropneumoniae* in bronchoalveolar fluid. BMC Microbiol. 2009; doi: 10.1186/1471-2180-9-195 10.1186/1471-2180-9-195 PMC2752462 19751522

[14] Stevenson A, Macdonald J, Roberts M (2003). Cloning and characterization of type 4 fimbrial genes from *Actinobacillus pleuropneumoniae*. Vet Microbiol.

[15] Boekema BK, Van Putten JP, Stockhofe-Zurwieden N, Smith HE (2004). Host cell contact-induced transcription of the type IV fimbria gene cluster of *Actinobacillus pleuropneumoniae*. Infect Immun.

[16] Labrie J, Pelletier-Jacques G, Deslandes V, Ramjjet M, Nash J (2010). Effects of growth conditions on biofilm formation by *Actinobacillus pleuropneumoniae*. Vet Res.

[17] Wu C, Labrie J, Tremblay YD, Haine D, Mourez M (2013). Zinc as an agent for the prevention of biofilm formation by pathogenic bacteria. J Appl Microbiol.

[18] Bello-Ortí B, Deslandes V, Tremblay YD, Labrie J, Howell KJ, Tucker AW, Maskell DJ, Aragon V, Jacques M (2015). Biofilm formation by virulent and non-virulent strains of *Haemophilus parasuis*. Vet Res.

[19] Costerton JW, Stewart PS, Greenberg EP (1999). Bacterial biofilms: a common cause of persistent infections. Science.

[20] Stoodley P, Sauer K, Davies DG, Costerton JW (2002). Biofilms as complex differentiated communities. Annu Rev Microbiol.

[21] Bjarnsholt T, Alhede M, Alhede M, Eickhardt-Sørensen SR, Moser C (2013). The in vivo biofilm. Trends Microbiol.

[22] Yang L, Liu Y, Wu H, Høiby N, Molin S (2011). Current understanding of multispecies biofilms. Int J Oral Sci.

[23] Fröls S (2013). Archaeal biofilms: widespread and complex. Biochem Soc Trans.

[24] Orell A, Fröls S, Albers SV (2013). Archaeal Biofilms: The Great Unexplored. Annu Rev Microbiol.

[25] Serra DO, Richter AM, Klauck G, Mika F, Hengge R. Microanatomy at cellular resolution and spatial order of physiological differentiation in a bacterial biofilm. mBio. 2013; doi: 10.1128/mBio.00103-13 10.1128/mBio.00103-13 PMC3604763 23512962

[26] Standar K, Kreikemeyer B, Redanz S, Münter WL, Laue M, Podbielski A. Setup of an In Vitro Test System for Basic Studies on Biofilm Behavior of Mixed- Species Cultures with Dental and Periodontal Pathogens. PLoS ONE. 2010; doi: 10.1371/journal.pone.0013135 10.1371/journal.pone.0013135 PMC2948514 20957048

[27] Tremblay YD, Lamarche D, Chever P, Haine D, Messier S (2013). Characterization of the ability of coagulase-negative staphylococci isolated from the milk of Canadian farms to form biofilms. J Dairy Sci.

[28] Jensen HE, Gyllensten J, Hofman C, Leifsson PS, Agerholm JS (2010). Histologic and bacteriologic findings in valvular endocarditis of slaughter-age pigs. J Vet Diagn Invest.

[29] Wongnarkpet S, Pfeiffer DU, Morris RS, Fenwick SG (1998). An on-farm study of the epidemiology of *Actinobacillus pleuropneumoniae* infection in pigs as part of a vaccine efficacy trial. Prev Vet Med.

[30] O'Reilly T, Niven DF (1986). Defining the Metabolic and Growth Responses of Porcine Haemophili to Exogenous Pyridine Nucleotides and Precursors. J Gen Microbiol.

[31] Martin PR, Shea RJ, Mulks MH (2001). Identification of a Plasmid-Encoded Gene from *Haemophilus ducreyi* Which Confers NAD Independence. J Bacteriol.

[32] Assavacheep P, Rycroft AN (2012). Survival of *Actinobacillus pleuropneumoniae* outside the pig. Res Vet Sci.

[33] Opriessnig T, Giménez-Lirola LG, Halbur PG (2011). Polymicrobial respiratory disease in pigs. Anim Health Res Rev.

[34] Biyikoglu B, Ricker A, Diaz PI (2012). Strain-specific colonization patterns and serum modulation of multi-species oral biofilm development. Anaerobe.

[35] Bridier A, Sanchez-Vizuete MP, Le Coq D, Aymerich S, Meylheuc T (2012). Biofilms of a *Bacillus subtilis* Hospital Isolate Protect *Staphylococcus aureus* from Biocide Action. PLoS ONE.

[36] de Chávez Paz LE (2012). Development of a Multispecies Biofilm Community by Four Root Canal Bacteria. J Endod..

[37] Lopes SP, Ceri H, Azevedo NF, Pereira MO (2012). Antibiotic resistance of mixed biofilms in cystic fibrosis: impact of emerging microorganisms on treatment of infection. Int J Antimicrob Agents.

[38] Stewart CR, Muthye V, Cianciotto NP. *Legionella pneumophila* Persists within Biofilms Formed by *Klebsiella pneumoniae*, *Flavobacterium* sp., and *Pseudomonas fluorescens* under Dynamic Flow Conditions. PLoS ONE. 2012; doi: 10.1371/journal.pone.0050560. 10.1371/journal.pone.0050560 PMC3503961 23185637

[39] Ryan RP, Fouhy Y, Garcia BF, Watt S, Niehaus K (2008). Interspecies signalling via the *Stenotrophomonas maltophilia* diffusible signal factor influences biofilm formation and polymyxin tolerance in *Pseudomonas aeruginosa*. Mol Microbiol.

[40] Almeida C, Azevedo N, Santos S, Keevil C, Vieira M (2011). Discriminating Multispecies Populations in Biofilms with Peptide Nucleic Acid Fluorescence In Situ Hybridization (PNA FISH). PLoS ONE.

[41] Schlafer S, Raarup MK, Wejse PL, Nyvad B, Städler BM (2012). Osteopontin Reduces Biofilm Formation in a Multi-Species Model of Dental Biofilm. PLoS ONE.

[42] Burmølle M, Thomsen TR, Fazli M, Dige I, Christensen L, Homøe P, Tvede M, Nyvad B, Tolker-Nielsen T, Givskov M, Moser C, Kirketerp-Møller K, Johansen HK, Høiby N, Jensen PØ, Sørensen SJ, Bjarnsholt T (2010). Biofilms in chronic infections -a matter of opportunity- monospecies biofilms in multispecies infections. FEMS Immunol Med Microbiol.

[43] Bertesteanu S, Triaridis S, Stankovic M, Lazar V, Chifiriuc MC, Vlad M, Grigore R (2014). Polymicrobial wound infections: Pathophysiology and current therapeutic approaches. Int J Pharm.

[44] Kaplan JB, Velliyagounder K, Ragunath C, Rohde H, Mack D (2004). Genes involved in the synthesis and degradation of matrix polysaccharide in *Actinobacillus actinomycetemcomitans* and *Actinobacillus pleuropneumoniae* biofilms. J Bacteriol.

[45] Ali Mohammed MM, Nerland AH, Al-Haroni M, Bakken V. Characterization of extracellular polymeric matrix, and treatment of *Fusobacterium nucleatum* and *Porphyromonas gingivalis* biofilms with DNase I and proteinase K. J Oral Microbiol. 2013; doi: 10.3402/jom.v5i0.20015 10.3402/jom.v5i0.20015 PMC3559756 23372876

[46] Dominiak D, Nielsen JN, Nielsen PH (2011). Extracellular DNA is abundant and important for microcolony strength in mixed microbial biofilms. Environ Microbiol.

[47] Jakubovics NS, Shields RC, Rajarajan N, Burgess JG (2013). Life after Death: The Critical Role of Extracellular DNA in Microbial Biofilms. Lett Appl Microbiol.

[48] Tang L, Schramm A, Neu T, Revsbech NP, Meyer RL (2013). Extracellular DNA in adhesion and biofilm formation of four environmental isolates: a quantitative study. FEMS Microbiol Ecol.

[49] Hathroubi S, Fontaine-Gosselin SE, Tremblay YDN, Labrie J, Jacques M (2015). Sub-inhibitory concentrations of penicillin G induce biofilm formation by field isolates of *Actinobacillus pleuropneumoniae*. Vet Microbiol.

[50] Olson ME, Ceri H, Morck DW, Buret AG, Read RR (2002). Biofilm bacteria: formation and comparative susceptibility to antibiotics. Can J Vet Res.

[51] Rajagopal R, Nair GK, Mini M, Joseph L, Saseendranath MR, John K (2013). Biofilm formation of *Pasteurella multocida* on bentonite clay. Iran J Microbiol.

[52] Romanò CL, De Vecchi E, Vassena C, Manzi G, Drago L (2013). A case of a late and atypical knee prosthetic infection by no-biofilm producer *Pasteurella multocida* strain identified by pyrosequencing. Pol J Microbiol.

