# Supplementary figures and images for: Auxotrophic Actinobacillus pleurpneumoniae grows in multispecies biofilms without the need for nicotinamide-adenine dinucleotide (NAD) supplementation
Source: BMC Microbiol. 2016 Jun 27;16:128. doi: 10.1186/s12866-016-0742-3 (PMC4924255; doi:10.1186/s12866-016-0742-3)

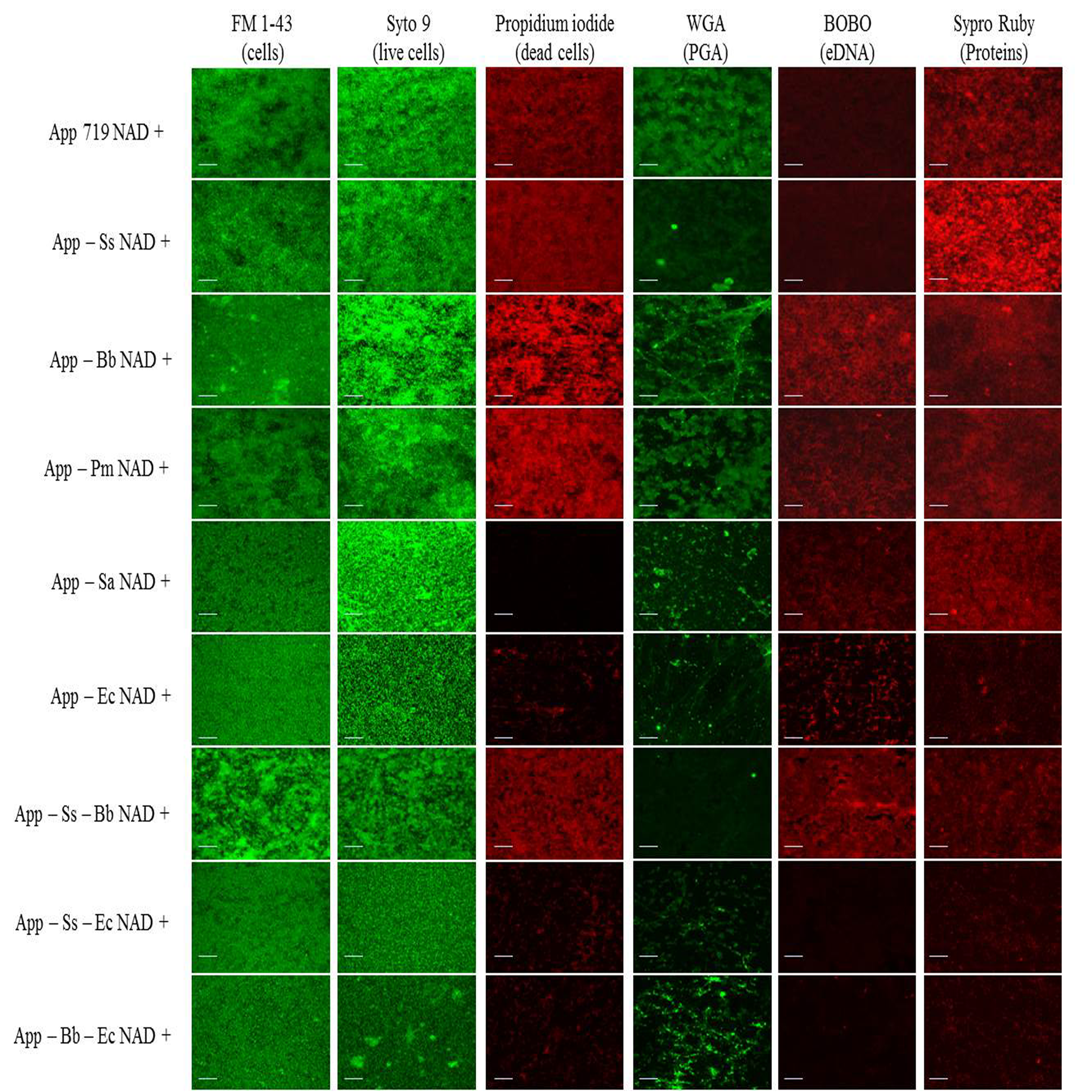

Supplement: Additional file 1: Figure S1. — CLSM of multi-species biofilms of A. pleuropneumoniae with the other swine pathogens with NAD supplementation. A. pleuropneumoniae, S. suis, B. bronchiseptica, P. multocida, S. aureus and E. coli grown in single, dual or triple-species biofilms in BHI media with NAD stained with FM 1-43, SYTO 9, propidium iodide, wheat-germ agglutinin (WGA)-Oregon green, BOBO-3, and SYPRO Ruby (all from Invitrogen, Eugene, OR). PGA: poly-N-acetylglucosamine; eDNA: extracellular DNA; App: A. pleuropneumoniae; Ss: S. suis; Bb: B. bronchiseptica; Pm: P. multocida; Sa: S. aureus; Ec: E. coli. Scale bar 30 μm (TIF 37984 kb) [file 12866_2016_742_MOESM1_ESM.tif]
